# Hematological and biochemical abnormalities associated with severe forms of COVID-19: A retrospective single-center study from Morocco

**DOI:** 10.1371/journal.pone.0246295

**Published:** 2021-02-04

**Authors:** Aziza Kantri, Jihane Ziati, Mohamed Khalis, Amal Haoudar, Karim El Aidaoui, Youssef Daoudi, Inas Chikhaoui, Khalid El Yamani, Mohammed Mouhaoui, Jalila El Bakkouri, Nezha Dini, Mohammed Mahi, Abdelhamid Naitlho, Abdelkrim Bahlaoui, Ahmed Bennana, Mustapha Noussair, Lahcen Belyamani, Chafik El Kettani

**Affiliations:** 1 Sheikh Khalifa International University Hospital, Faculty of Medicine, Mohammed VI University of Health Sciences, Casablanca, Morocco; 2 International School of Public Health, Mohammed VI University of Health Sciences, Casablanca, Morocco; 3 Research Methodology Support Unit, Mohammed VI University of Health Sciences, Casablanca, Morocco; 4 Faculty of Medicine and Pharmacy, Hassan II University, Casablanca, Morocco; 5 Faculty of Medicine and Pharmacy, Mohammed V University, Rabat, Morocco; 6 Military Training Hospital Mohammed V, Mohammed V University, Rabat, Morocco; Tekirdag Namik Kemal University, TURKEY

## Abstract

Since December 2019, the coronavirus disease (COVID-19) pandemic has catapulted the world into a marked health crisis, with over 29 million cases and >930,000 deaths. To better detect affected individuals at an early stage and stop disease progression to an advanced stage, several studies have been conducted to identify the clinical, biological, and radiological characteristics of COVID-19. This study aimed to enrich the literature by critically analyzing the clinical and biological characteristics of 134 patients from the North African Mediterranean region, including numerous genetic, epigenetic, and environmental factors that may influence disease evolution. This single-center retrospective study included all patients older than 18 years confirmed to have COVID-19 and hospitalized at the Cheikh Khalifa University Hospital affiliated with Mohammed VI University of Health Sciences, Casablanca, Morocco. Clinical, demographic, and biological data were analyzed in a cohort of severe and non-severe patients. Univariate analysis was performed to identify factors predictive of severity. There were 134 patients: the median age was 53 years, and 54.5% were male. Of these, 89 had mild to moderate disease; 45 had severe to critical disease, of which 14 died and 31 survived. Advanced age, presence of comorbidities, male sex, and infection in ethnic or family groups were risk factors for progression to severe disease. The presence of abnormalities in the following parameters were strongly associated with progression to severe disease: white blood cells (WBC), neutrophils, lymphocytes, C-reactive protein (CRP), procalcitonin, D-dimers, lactate dehydrogenase (LDH), ferritin, creatinine, aspartate aminotransferase (ASAT), and alanine aminotransferase (ALAT) during both admission and hospitalization. Based on these results and an extensive literature review, we recommend that clinicians closely monitor the biological parameters identified herein and perform immunological and genetic studies.

## Introduction

Coronavirus disease (COVID-19) is an emerging disease that is spreading rapidly worldwide and threatens the biosecurity of all countries [1, 2], with the number of cases exceeding 29,000,000 and a death toll of more than 930,000. The United States of America and India are the two most affected countries as of September 17, 2020, with a death toll of 199,746 in the USA and 83,198 in India.

In March 2020, the first case of confirmed COVID-19 was recorded in the kingdom of Morocco; the patient, who presented with acute pneumonia, was an imported case from Europe. As of September 17, 2020, the number of cases confirmed in the different regions of our country was 92,016, including 1,686 deaths, the majority of which were diagnosed after the deconfinement in June 2020.

This rapid viral spread has prompted the publication of numerous studies to identify clinical, biological, radiological, and genetic predictors for the progression to severe and fatal forms of the disease [3]. Recognition of these predictors will make it possible to stratify the risk and direct the intervention studies to target patients at risk of worsening and progression to death. Moreover, such predictors would also allow for the optimized allocation the human and technical resources for management. Demographic (advanced age, male sex), clinical (comorbidities, acute respiratory distress syndrome [ARDS]), and radiological predictors have been extensively detailed in different studies [3–5]. Biological (lymphopenia, hyperferritinemia, serum C-reactive protein [CRP] levels) predictors [3, 6] have been reported but remain mostly undescribed in the North African region.

Our study enriches these data by reporting the experience in our developing country, which undertook confinement measures at an early stage of the crisis, thereby impacting the evolution of this pathology. The objective of our study was to describe the hematological and biochemical abnormalities in Moroccan patients with COVID-19 and to identify the parameters that can help distinguish those likely to develop severe COVID-19.

## Methods

### Study design

This single-center, retrospective, observational study was approved by the institutional scientific and ethics committees of Cheikh Khalifa International University Hospital and Mohammed VI University of Health Sciences (UM6SS), and consent was not required (CE_UM6SS/1/06/2020-April 3th 2020). This study describes the demographic characteristics, clinical presentation, laboratory findings, and outcomes of incident cases of COVID-19 admitted to the intensive care unit (ICU) from March 18, 2020, the date of the first confirmed case in our hospital, until May 20, 2020. This time frame was chosen to have a minimum follow-up of 15 days for all patients. The last patient in the series was admitted on April 20, 2020.

### Participants and eligibility criteria

Only patients with confirmed severe acute respiratory syndrome coronavirus 2 (SARS-CoV-2) infection were included. A confirmed case of COVID-19 was defined by a positive result on a reverse-transcriptase polymerase chain reaction (RT-PCR, Kit Genfinder) assay using a nasopharyngeal swab specimen. Only laboratory-confirmed cases were included [7]. A total of 146 patients were recruited, excluding patients under 18 years of age; ultimately, 134 patients were selected and separated into two groups: 45 severe patients and 89 non-severe patients. Severely ill patients were defined as those admitted to the ICU with one of the following signs: respiratory rate more than 30 breaths/min, oxygen saturation less than 93% at room air, ARDS, or requirement of mechanical ventilation [8]. Non-severe patients were those with mild or moderate forms of COVID-19 according to World Health Organization criteria [8].

### Data collection

Electronic medical record data were collected using institutional software (DxCare SIH-HCK and LIMS SGL-HCK). Data collected included demographic, clinical, and biological factors, as well as complications at admission and during the hospital stay. Any missing or uncertain data were collected and clarified through direct communication with the relevant health care providers and family members of patients.

### Statistical analysis

Continuous variables were presented as means (standard deviation [SD]) if they were normally distributed or as medians (interquartile range [IQR]) if they were not normally distributed; categorical variables were presented as counts (%). The Kolmogorov-Smirnov test was used to assess the normality of distribution of continuous variables. Baseline demographic, clinical, and biological characteristics were compared among severe and non-severe patients. To compensate for the lack of data on certain biological parameters at a given time due to patient discharge or death, the parameters at admission, nadir, and peak during hospitalization were compared. Nadir and peak hematological parameters were obtained by following patients from admission to their last blood test. To compare differences between the two groups, we used the Student’s t-test (parametric distribution) or Mann-Whitney’s U test (nonparametric distribution) for continuous variables and the chi-squared or Fisher’s exact test for categorical variables. For all tests, a two-tailed α below 0.05 was considered statistically significant (P-value). Statistical analyses were performed using SPSS version 26.0 (IBM SPSS).

## Results

The number of confirmed COVID-19 patients was 134; 89 (66.4%) were admitted to single bedroom, and 45 (33.5%) patients with severe to critical disease required treatment in the ICU. The latter were distributed as follows: 31 survivors and 14 non-survivors.

The mean age of the patients was 53 years (36–64); men were predominant (73 [54.5%]). Slightly more than half of the patients (68 [50.7%]) had comorbidities, with hypertension (36 [26.9%]) being the most common, followed by diabetes (19 [14.2%]) and coronary heart disease (16 [11.9%]). The time from symptom onset to hospital admission was 7 days (IQR, 3.0–7.2). In addition, 68 patients (65.4%) were infected via transmission through an ethnic or family cluster. Fever (61 [45.5%]) was the most frequently observed symptom, followed by dry cough (59 [44%]) and dyspnea (39 [29%]). The other symptoms (myalgia, asthenia, headache, anosmia, ageusia, and digestive signs) were observed in our series less frequently (Table 1).

**Table 1:** Demographic and clinical characteristics of patients.

Characteristics	Total (n = 134)	Severe (n = 45)	Non-severe (n = 89)	P-value
**Age (years)**^1^	53 [36–64]	64 [57–74]	42 [29–56]	<0.001
**Sex**^2^				
Male	73 (54.5%)	35 (77.8%)	38 (42.7%)	
Female	61 (45.5%)	10 (22.2%)	51 (57.3%)	<0.001
**Time from illness onset to admission**^1^	7 [3.0–7.2]	3 [2–7]	7 [4–8]	0.155
**Cluster**^2^	75 (56%)	31 (68.9%)	44 (49.4%)	
Yes	59 (44%)	14 (31.1%)	45 (50.6%)	0.032
No				
**Comorbidities**^2^	19 (14.2%)	12 (26.7%)	7 (7.9%)	
Diabetes*	36 (26.9%)	22 (48.9%)	14 (15.7%)	0.003
HTA*	16 (11.9%)	13 (28.9%)	3 (3.4%)	<0.001
Cardiac disease	10 (7.5%)	5 (11.1%)	5 (5.6%)	
Asthma	7 (5.2%)	2 (4.4%)	5 (5.6%)	<0.001
Tobacco use	3 (2.2%)	3 (6.7%)	0	
Chronic kidney disease	2 (1.5%)	1 (2.2%)	1 (1.1%)	0.303
Malignancy	1 (0.7%)	1 (2.2%)	0	1.000
Chronic liver disease	1 (0.7%)	1 (2.2%)	0	0.036
Ischemic stroke				1.000
**Signs and symptoms**^2^				
Asthenia	25 (18.7%)	22 (49%)	3 (3.4%)	-
Fever	61 (45.5%)	27 (60%)	34 (38.2%)	-
Headache	21 (15.7%)	5 (11%)	16 (18%)	-
Myalgia	30 (22.4%)	7 (15.6%)	23 (25.8%)	-
Rhinitis	8 (6%)	3 (6.7%)	5 (5.6%)	-
Pharyngalgia	10 (7.5%)	5 (11%)	5 (5.6%)	-
Ageusia	28 (21%)	7 (15.6%)	23 (25.8%)	-
Anosmia	25 (18.7%)	5 (11%)	20 (22.5%)	-
Dry cough	59 (44%)	24 (53.3%)	35 (39.3%)	-
Dyspnea	39 (29%)	25 (55.6%)	14 (15.7%)	-
Chest pain	5 (3.7%)	2 (4.4%)	3 (3.4%)	-
Abdominal pain	18 (13.4%)	5 (11%)	13 (14.6%)	-
Diarrhea	29 (21.6%)	11 (24.4%)	18 (20.2%)	-
Vomiting	22 (16.4%)	6 (13.3%)	16 (18%)	-
Asymptomatic status	20 (15%)	1 (2.2%)	19 (21.3%)	-
**Complications**^2^				
ARDS	13 (9.7%)	13 (28.9%)	0	-
Extrarenal epuration	8 (6%)	8 (17.8%)	0	-
Arrythmia	4 (2.9%)	4 (8.8%)	0	-
Pneumothorax	2 (1.5%)	2 (4.4%)	0	-
Thromboembolic disease	4 (2.9%)	4 (8.8%)	0	-
Septic shock	1 (0.01%)	1 (2.2%)	0	-
Death	14 (10.4%)	14 (31.9%)	0	-
**Causes of death**^2^				
ARDS + AKF	8 (5.9%)	8 (17.8%)	0	-
ARDS	4 (3%)	4 (8.9%)	0	-
Septic shock	1 (0.7%)	1 (2.2%)	0	-
Decompensated cirrhosis	1 (0.7%)	1 (2.2%)	0	-

P values were calculated by the Student’s t-test or Mann-Whitney U test for continuous variables and the chi-squared test or Fisher’s exact test for categorical variables.

^1^Data are shown as medians (quartiles).

^2^Data are shown as frequencies (%).

ARDS: acute rrespiratory distress syndrome.

Most of the patients received antiviral therapy, including hydroxychloroquine (127 patients [95%]) and azithromycin (127, [95%]), with no difference between the two groups. Severe patients received lopinavir/ritonavir (11, [24.4%]). Five patients in the severe group received tocilizumab (11.1%), one of whom survived after 7 days of invasive mechanical ventilation. The majority of patients received low-molecular-weight heparin: 95% of patients with severe disease received a curative dose and 76% of patients with moderate disease received a preventive dose. Corticotherapy was used in most critical cases (31% of severe patients).

In the severe group, 14 deaths (31.9%) occurred, of which 11 (24.4%) patients underwent invasive mechanical ventilation and 3 (6.6%) had sudden cardiorespiratory arrest under non-invasive ventilation. The causes of death were predominantly due to progression to severe ARDS alone (4 [8.9%]) or associated with acute kidney injury (8 cases [17.7%]), septic shock (1 [2.2%]), and cirrhotic decompensation (1 [2.2%]). Other complications the severe patients included thromboembolic events (4 [8.9%]), arrhythmia (4 [8.9%]) and pneumothorax (2 [4.4%]).

**Fig 1:**
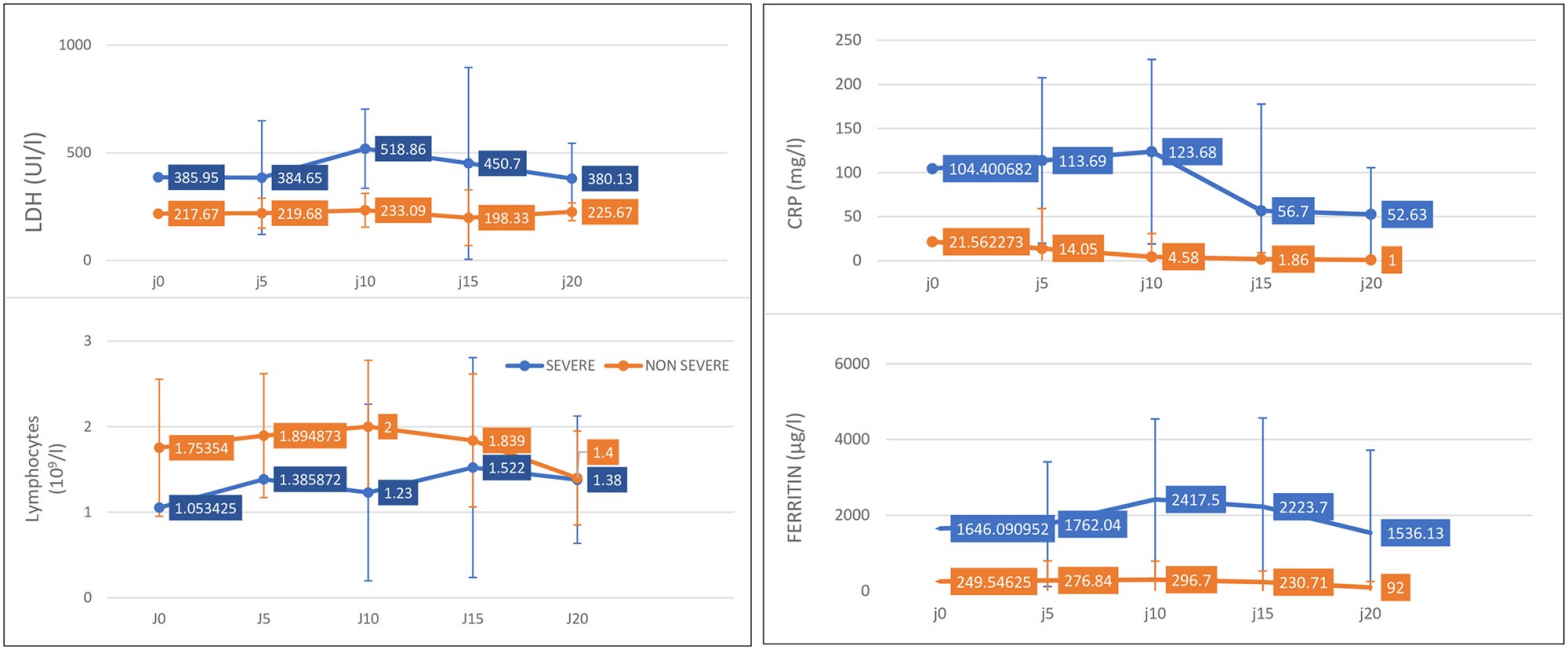
Temporal changes in laboratory markers from illness onset in patients hospitalized with COVID-19.

In univariate analysis, severe patients had significantly higher median age (64 years, [IQR, 57–74], P<0.001) and more comorbidities, dominated by hypertension (22 [8.9%] versus 14 [15.7%], P<0.000), diabetes (12 [26.7%] versus 7 [7.9%], P<0.003) and cardiac disease (13 [28.9%] versus 3 [3.4%], P<0.001). Transmission via ethnic or family clusters also appears to favor progression to severe disease. High levels of white blood cells (WBC), neutrophils, CRP, procalcitonin, D-dimers, lactate dehydrogenase (LDH), ferritin, creatinine, alanine aminotransferase (ALAT), and aspartate aminotransferase (ASAT), both on admission and during hospitalization, were strongly associated with progression to severe forms (Tables 2 and 3). Lymphopenia and a tendency toward leukopenia and neutropenia also significantly favored severity (Tables 2 and 3). By following the kinetics of the biological assessment over the entire period of hospitalization, it was observed that the difference was significant between the two groups; moreover, the levels of LDH, ferritin, and CRP in severe patients began to regress from the 10th day of hospitalization in the ICU (Fig 1).

**Table 2:** Laboratory findings of patients on admission.

	Total	Severe	Non-severe	P-value
	n = 134	n = 45	n = 89	
**Hemoglobin (g/l) n = 131**	13.9 [12.8–15]	14.1 [12.6–14.9]	13.7 [12.8–15.07]	0.433
**Platelets (10**^**9**^**/l) n = 131**	224 [181–293]	214 [169–314]	228.5 [187–289]	0.565
**White blood cells (10**^**9**^**/l) n = 131**	6.31 [4.7–7.35]	6.64 [5.41–8.22]	5.78 [4.46–7.24]	0.051
**Neutrophils (10**^**9**^**/l) n = 131**	4.03 [2.66–5.44]	4.85 [3.45–6.84]	3.69 [2.28–4.89]	<0.001
**Lymphocytes (10**^**9**^**/l) n = 131**	1.41 [0.96–2.01]	0.995 [0.71–1.31]	1.65 [1.21–2.14]	0.003
**CRP (mg/l) n = 132**	11.9 [2.52–68]	86 [21–148]	3.85 [1.41–20.83]	<0.001
**Procalcitonin (ng/ml) n = 113**	0.05 [0.05–0.05]	0.08 [0.05–0.35]	0.05 [0.01–0.005]	0.001
**D-dimer (μg/l) n = 85**	0.54 [0.3–0.92]	0.828 [0.54–2.44]	0.431 [0.283–0.860]	0.001
**Urea (mmol/l) n = 128**	4 [3.2–5.3]	5.1 [4–6.6]	3.5 [3–4.8]	0.510
**Creatinine (μmol/l) n = 128**	72 [63–84]	83 [71–106]	68 [62–77]	0.001
**Ferritin (μg/l) n = 93**	160.4 [64.9–450]	645.7 [383.8–2289]	130 [42–229]	<0.001
**ALAT(UI/l) n = 105**	22 [15–35]	35 [20–55]	20 [17–26]	0.001
**ASAT (UI/l) n = 105**	23 [18.5–33]	34 [20–55.5]	19 [14–29]	0.012
**LDH (UI/l) n = 89**	228 [178.5–281]	330 [253–399]	215 [164–252]	<0.001
**Troponin (ng/ml) n = 86**	0,003 [0,002–0,062]	0,005 [0,003–0,107]	0,002 [0,001–0,004]	0,26

P values were calculated by the Student’s t-test or Mann-Whitney U test for continuous variables and chi-squared test or Fisher’s exact test for categorical variables.

Data are shown as medians [quartiles].

**Table 3:** Blood profile during inpatient stay (nadir and peak of laboratory findings).

		Total	Severe	Non-severe	P-value
		n = 134	n = 45	n = 89	
***NADIR***	**Hemoglobin (g/l) n = 104**	12.9 [11.7–13.8]	12,2 [10.5–13.3]	13.2 [12.4–14.3]	0.00
	**White blood cells (10**^**9**^**/l) n = 104**	5.85 [4.9–7.4]	7.13 [5.6–9.3]	5.54 [4.6–6.28]	0.001
	**Neutrophils (10**^**9**^**/l) n = 104**	3.3 [2.59–5.37]	5.25 [3.2–7.4]	3 [2.39–3.8]	0.001
	**Lymphocytes (10**^**9**^**/l) n = 104**	1.42 [0.71–1.91]	0.71 [0.43–1.26]	1.71 [1.31–2.2]	0.001
***PEAK***	**White blood cell (10**^**9**^**/l) n = 105**	6.5 [5.6–9.52]	9.5 [6.6–18.7]	5.97 [5.35–7.13]	0.001
	**Neutrophils (10**^**9**^**/l) n = 105**	4.19 [3.06–6.46]	7.31 [4.7–15.5]	3.44 [2.35–4.28]	0.001
	**CRP (mg/l) n = 91**	12.9 [2–90.7]	90 [41–246]	2.6 [0.9–9.8]	0.001
	**Procalcitonin (ng/ml) n = 66**	0.1 [0.05–1.16]	0.5 [0.11–3.7]	0.05 [0.05–0.05]	0.001
	**D-dimer (μg/l) n = 90**	0.57 [0.32–1.5]	1.10 [0.58–4.04]	0.38 [0.216–0.613]	0.004
	**Creatinine (μmol/l) n = 93**	79 [65–103]	105 [79–281]	66 [61–80]	0.001
	**Urea (mmol/l) n = 93**	5 [3.51–7.8]	7.8 [5.5–1.23]	3.7 [3.3–4.5]	0.721
	**Ferritin (μg/l) n = 95**	273 [75–1760]	2,151 [849–3054]	92.07 [28–245]	0.001
	**ALAT (UI/l) n = 79**	43 [23–63]	51 [33–110]	32 [19–53.7]	0.001
	**ASAT (UI/l) n = 79**	36 [24–53]	34 [47–100]	26.5 [19–53.7]	0.017
	**LDH (UI/l) n = 93**	249 [185–380.5]	409 [228–523]	218 [178.7–301]	0.001
	**Troponin (ng/ml) n = 83**	0.005 [0.003–0.034]	0.03 [0.007–0.17]	0.03 [0.007–0.17]	0.002

P values were calculated by the Student’s t-test or Mann-Whitney U test for continuous variables and the chi-squared test or Fisher’s exact test for categorical variables.

Data are shown as medians [quartiles].

## Discussion

SARS-CoV-2 is the third type of coronavirus detected in the last two decades after SARS-CoV-1 and Middle East respiratory syndrome (MERS)-CoV, identified in 2003 and 2012, respectively [9, 10]. SARS-CoV-1 infection caused the death of 774 people in 2002–2003; this infection was noted in 8,096 people during this period. MERS-COV was responsible for a localized epidemic in the Middle East in 2012. The case-fatality rate was 38%.

We described the clinico-biological profile of COVID-19 disease in the North African region using a sample of 134 patients. The demographic characteristics identified in our series support the data reported by several authors who have confirmed that advanced age is a factor that predisposes patients to COVID-19 and promotes progression to severe disease and death [11, 12]. For instance, Zhou et al. showed in their study that age over 50 years was strongly associated with the occurrence of ARDS and age over 65 years was associated with mortality [12]. However, advanced age was also reported as an important independent predictor of mortality in SARS and MERS [13, 14]. The frequent age-related comorbidities observed in our patients are severity and prognostic factors that have been demonstrated by a large number of studies, such as those by Zhou et al and Wu et al, hypertension and diabetes were significantly associated with the occurrence of ARDS in a multivariate analysis and with the occurrence of mortality in a univariate analysis [11, 12]. The reason for the association between infection-related mortality (particularly viral infections) and age may be impaired cellular immune function and a longer duration of inflammation in elderly people [11]. Male sex has also been reported as a factor influencing the severity of COVID-19 by most authors; this was also confirmed in our series [15]. In contrast, a team from Iran showed that sex may not be a factor promoting aggravation [16]. Furthermore, transmission via ethnic or family clusters, which was noted in our series (68 [65.4%]), raises suspicion of a genetic predisposition to the disease, which needs to be identified by in-depth immunological and genetic studies. The clinical signs presented by our patients, as well as their frequencies and rates, were similar to those in other series [3].

It is evident that COVID-19 disease is associated with significant morbidity, particularly in patients with chronic diseases, at least one-fifth of whom require supportive care in medical ICUs [17], which are particularly limited in most developing countries such as those in Africa. Moreover, despite the implementation of optimal supportive interventions, the inpatient mortality rate remains above 1.4%, reaching 6.4% in the population aged over 60 years old [18, 19].

The biological profile of our patients is similar to what has been described in the literature, with the presence of lymphopenia in severe patients upon admission and an aggravation of lymphopenia during their stay. Several hypotheses have been raised to understand the pathogenesis of lymphopenia in the context of SARS-CoV-2 infection. A Chinese study details the characteristics of the hemogram and lymphocyte subpopulations in 166 patients with the non-severe form and 286 with the severe form. Severe patients had a significantly increased neutrophil/lymphocyte ratio and elevated markers of inflammation (CRP, ferritin, interleukin-6, interleukin-8, and interleukin-10). In addition, there was an imbalance in the lymphocyte immune response in severe patients, who had more CD4 lymphopenia, more CD4-naïve cells and CD4 suppressor T cells, and fewer CD4 memory cells and regulatory T cells, compared to that in non-severe patients [20]. Rodriguez et al. have suggested that COVID-19 may act on lymphocytes, particularly T cells, possibly depleting CD4 and CD8 cells [15].

The viral particles spread through the respiratory mucosa, first using the ACE2 receptor at the level of ciliated bronchial epithelial cells and then infecting other cells. This induces a cytokine storm in the body and generates a series of immune responses, which cause changes in peripheral WBCs and immune cells such as lymphocytes [21]. This notion has been demonstrated by Henry et al. in their meta-analysis: the number of lymphocytes, particularly CD4 lymphocytes, can serve as a biological predictor of severity and mortality; in the case of COVID-19, they also reported the hypothesis that survival may depend on the ability to restore lymphocytes that are killed by the virus [22]. The same authors also reported significant increases in ferritin and CRP levels in patients with suspected severe COVID-19, consistent with the current findings.

The increase in CRP levels reflects the extent of the systemic inflammatory syndrome seen in severe forms of the disease, which is accompanied by a massive release of inflammatory cytokines creating a "cytokine storm" responsible for acute tissue damage with the onset of severe ARDS and subsequent multi-systemic failure [22].

The increase in LDH levels observed in our series is consistent with the findings of Liu who correlated LDH, lymphocyte, neutrophil, and CRP abnormalities with severe COVID pneumonia [23]. The elevated ferritin levels are probably due to secondary phagocytic lymphohistiocytosis and severe cytokine release syndrome [24, 25]. An elevated D-dimer level was also significantly related to severity in our series and was the cause of coagulopathy with thromboembolic complications in 2.9% of our patients. Anemia and thrombocytopenia were not common in our series. Myocardial damage, as revealed biologically by high troponin and creatine phosphokinase levels indicative of viral myocarditis, which has been described by others, was not observed in our severe patients [26, 27].

In contrast, the evolution during the stay was marked by the significantly stark difference of the median nadir of WBC, lymphocytes, and Neutrophils, as well as the median peak of WBC, neutrophil, CRP, procalcitonin, ferritin, LDH, D-dimer, ASAT, ALAT, creatinine, and urea levels. This demonstrates the occurrence of complications of the severe form of COVID-19 including the following: bacterial superinfection, severe ARDS secondary to the cytokine storm, thromboembolic disease, and organ dysfunction (which is believed to be multifactorial [comorbidities, the cytokine storm of COVID-19, the resuscitation environment]), leading to death. This has been confirmed by several authors [3–29].

The limitations of our study were as follows: the single-center retrospective study design, which increases the chance of selection bias and impacts the generalizability of data; the absence of evaluation of immunological parameters (CD4, CD8, interleukin-6, interleukin-8, interleukin-10) studied by the other teams, which could help us to properly analyze the inflammatory characteristics of our patients; the small sample size; and missing data from some paucisymptomatic patients and patients who died at a given time. This limits the in-depth statistical analysis needed to stratify the maximum risks associated with the pathology.

## Conclusion

We have investigated multiple studies, the majority of which originated in China, which can be compared to that of other populations to detect clinical and biological characteristics of COVID-19 that may be influenced by genetic, epigenetic, and environmental factors. For this reason, we recommend that clinicians closely monitor the biological parameters in hospitalized patients with respiratory distress related to COVID-19 and that further genetic and virology studies be conducted to properly control the risk related to the disease.

## Supporting information

S1 Data (XLSX)
